# Supplementary material for: Investigation of Bemethyl Biotransformation Pathways by Combination of LC–MS/HRMS and In Silico Methods
Source: Int J Mol Sci. 2021 Aug 21;22(16):9021. doi: 10.3390/ijms22169021 (PMC8396642; doi:10.3390/ijms22169021)
Supplement: Supplementary file 1 [file ijms-22-09021-s001.zip › ijms-1325485-supplementary.pdf]

Raw LC–MS/HRMS data

Bemethyl

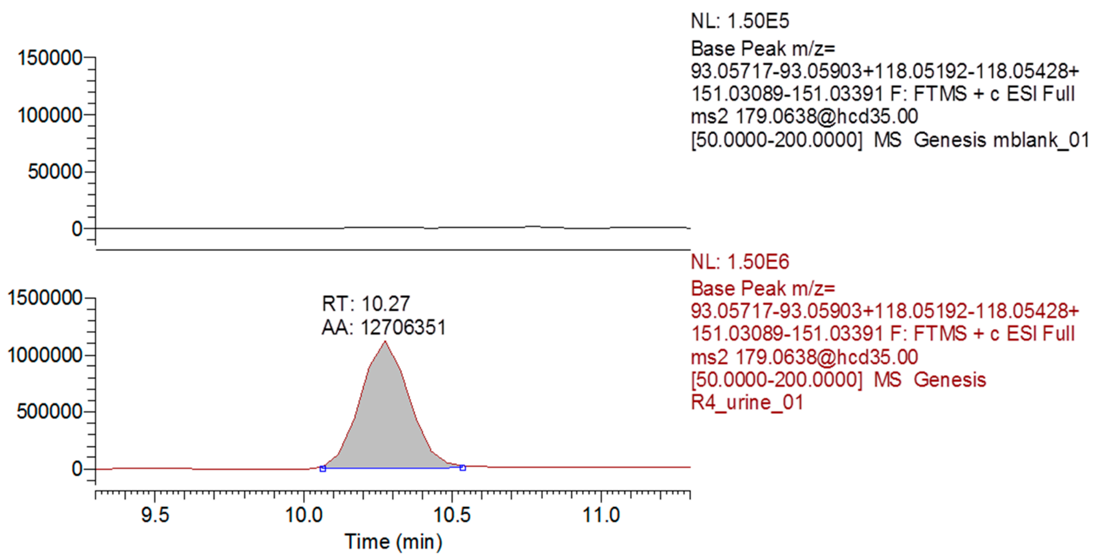

Figure S1. Reconstructed ion chromatogram of blank urine (top) and bemethyl (bottom) by product-ions (m/z) 151.0324, 93.0581, 118.0531.

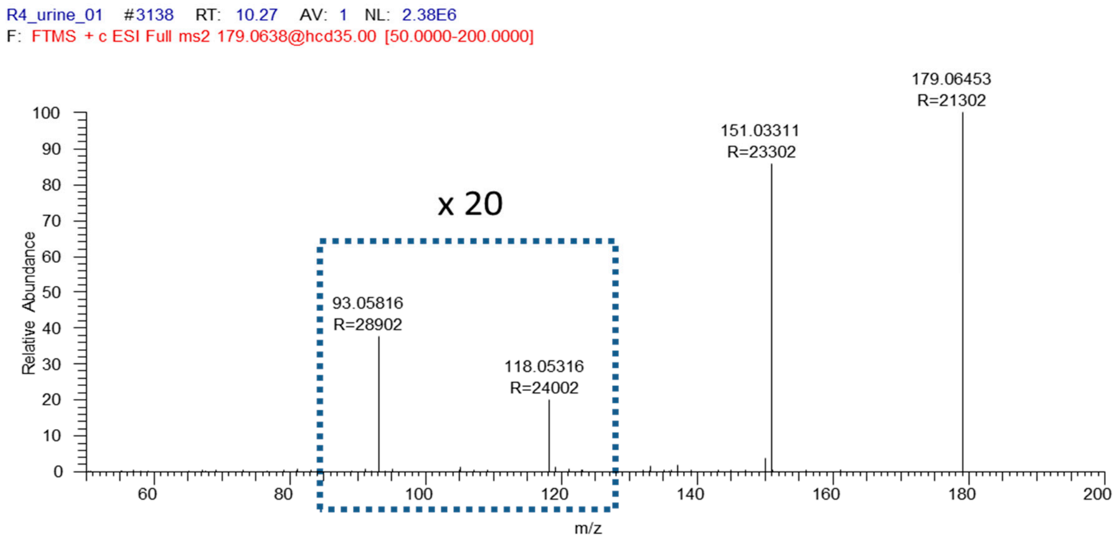

Figure S2. Mass-spectrum of bemethyl.

Table S1. Exact mass data for characteristic ions of bemethyl

**Compound**	**Parameter**	**Characteristic ions, *m/z***			
	Theoretical *m/z*	179.0638	151.0324	93.0581	118.0531
Bemethyl	Experimental *m/z*	179.06453	151.03311	93.05816	118.05316
	Mass error, ppm	-4.1	-4.7	-0.6	-0.5

M1a

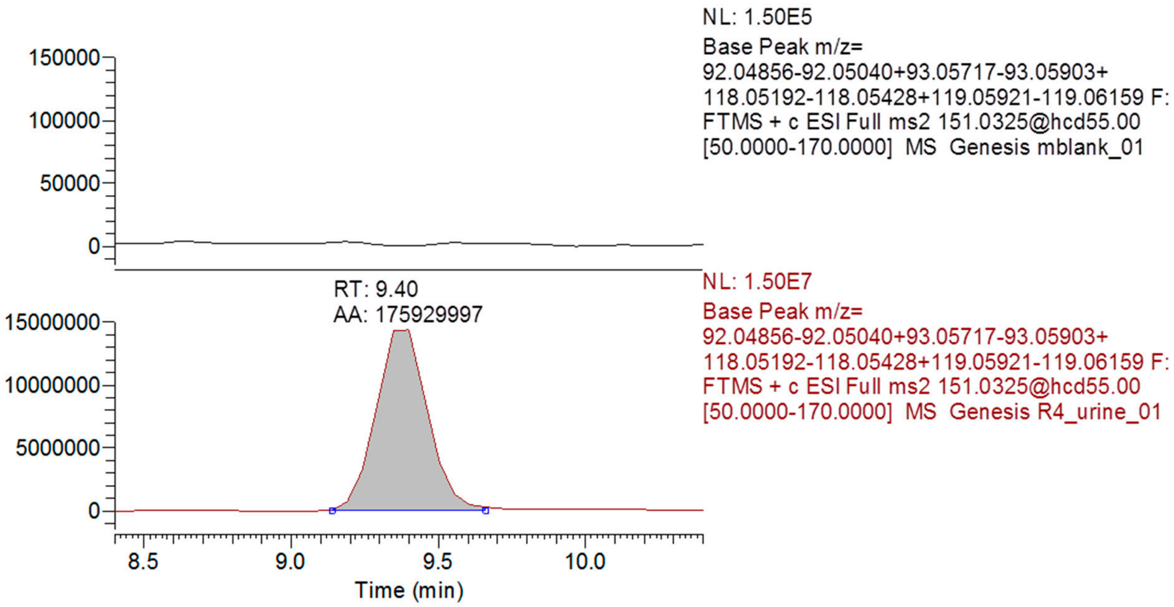

Figure S3. Reconstructed ion chromatogram of blank urine (top) and M1a (bottom) by product-ions (m/z) 92.04948, 93.0581, 118.0531, 119.0604.

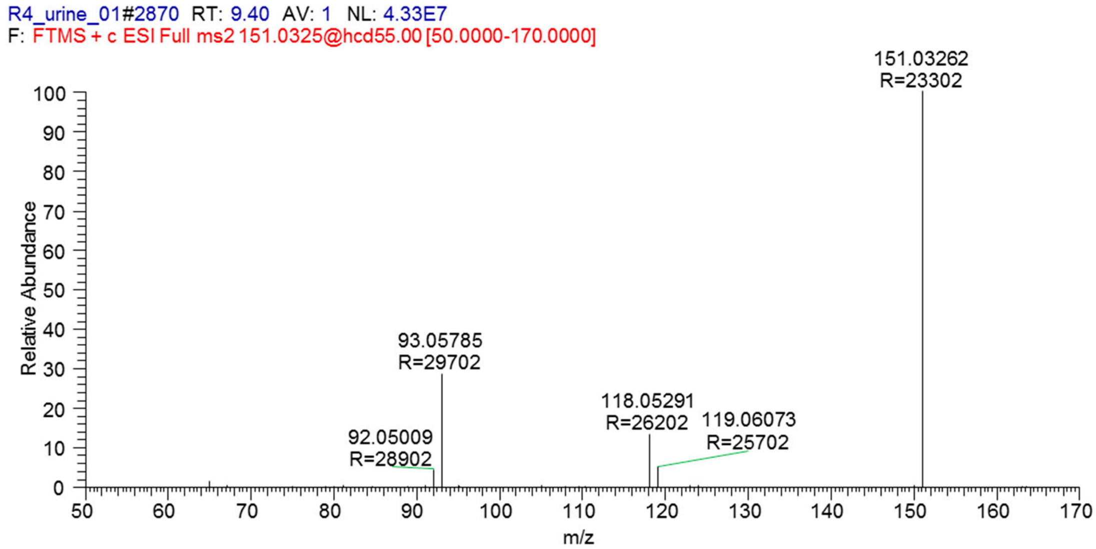

Figure S4. Mass-spectrum of M1a.

Table S2. Exact mass data for characteristic ions of M1a.

Compound	Parameter	Characteristic ions, *m/z*				
M1a	Theoretical *m/z*	151.0324	119.0604	118.0531	93.0581	92.0495
	Experimental *m/z*	151.03262	119.06073	118.05291	93.05785	92.05009
	Mass error, ppm	-1.5	-2.8	1.6	2.7	-6.4

M1b

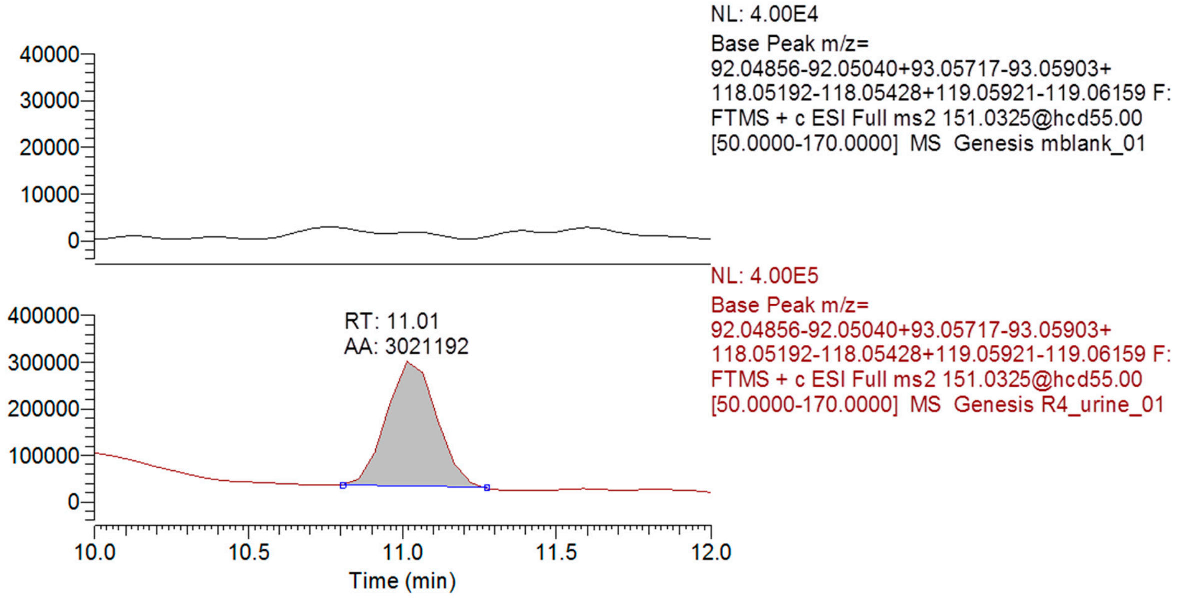

Figure S5. Reconstructed ion chromatogram of blank urine (top) and M1b (bottom) by product-ions (m/z) 92.04948, 93.0581, 118.0531, 119.0604.

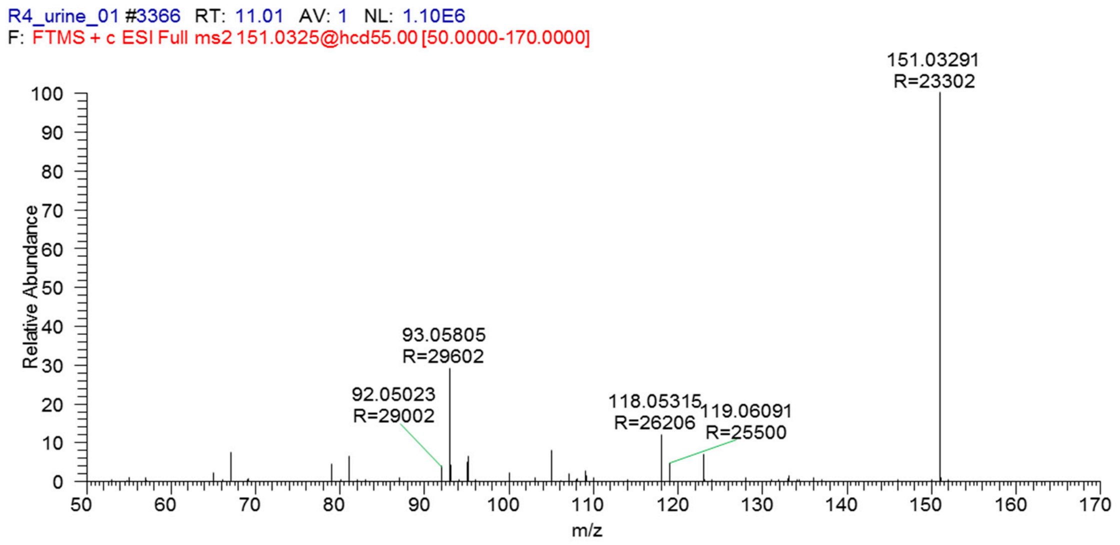

Figure S6. Mass-spectrum of M1b.

Table S3. Exact mass data for characteristic ions of M1b.

Compound	Parameter	Characteristic ions, *m/z*				
M1b	Theoretical *m/z*	151.0324	119.0604	118.0531	93.0581	92.0495
	Experimental *m/z*	151.03291	119.06091	118.05315	93.05805	92.05023
	Mass error, ppm	-3.4	-4.3	-0.4	0.5	-7.9

M2a

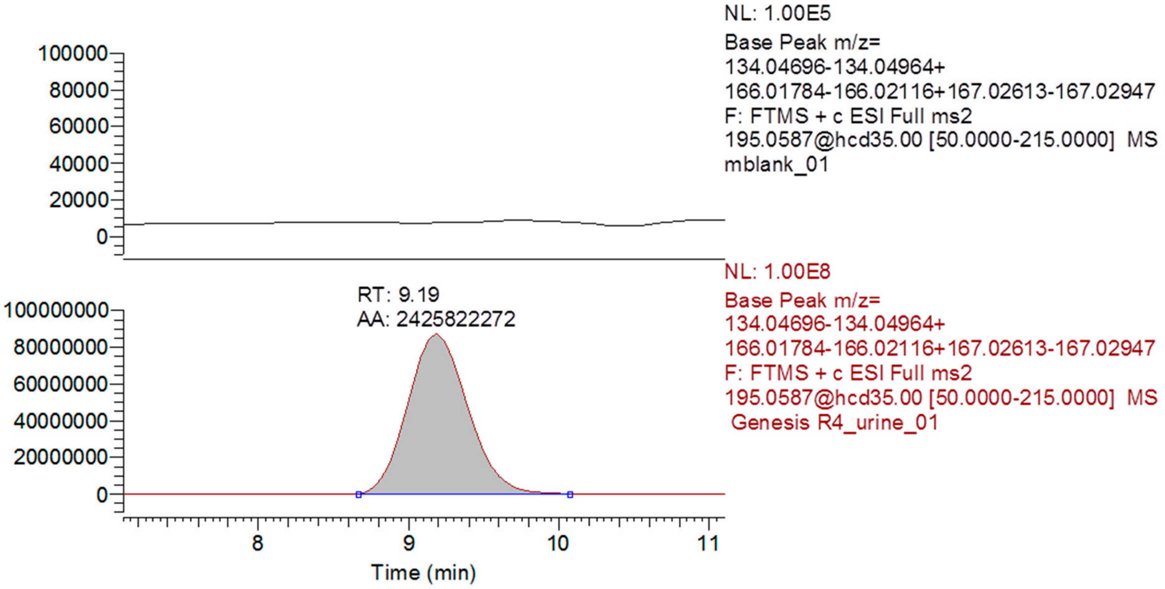

Figure S7. Reconstructed ion chromatogram of blank urine (top) and M2a (bottom) by product-ions (m/z) 134.0483, 166.0195, 167.0278.

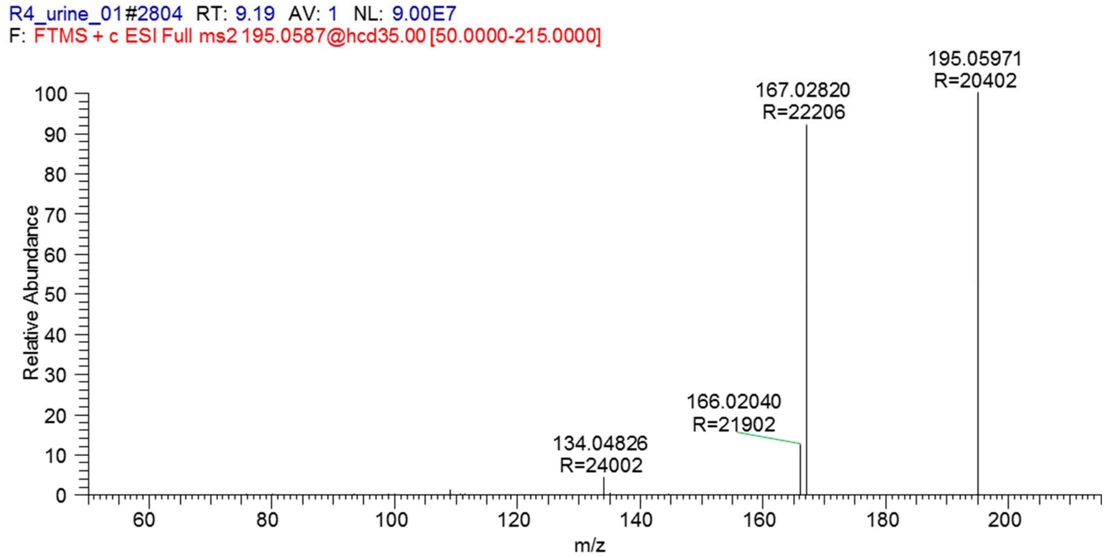

Figure S8. Mass-spectrum of M2a.

Table S4. Exact mass data for characteristic ions of M2a.

Compound	Parameter	Characteristic ions, *m/z*			
M2a	Theoretical *m/z*	195.0587	167.0279	166.0195	134.048
	Experimental *m/z*	195.05971	167.02820	166.02040	134.04826
	Mass error, ppm	-5.2	-1.8	-5.4	-1.9

M2b

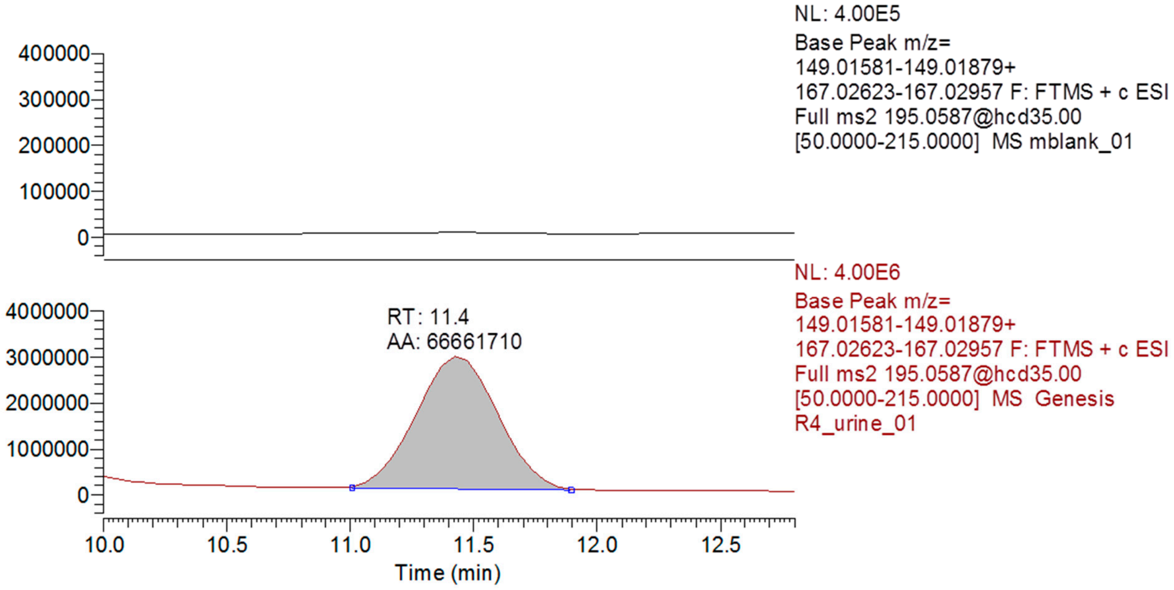

Figure S9. Reconstructed ion chromatogram of blank urine (top) and M2b (bottom) by product-ions (m/z) 149.0173, 167.0279.

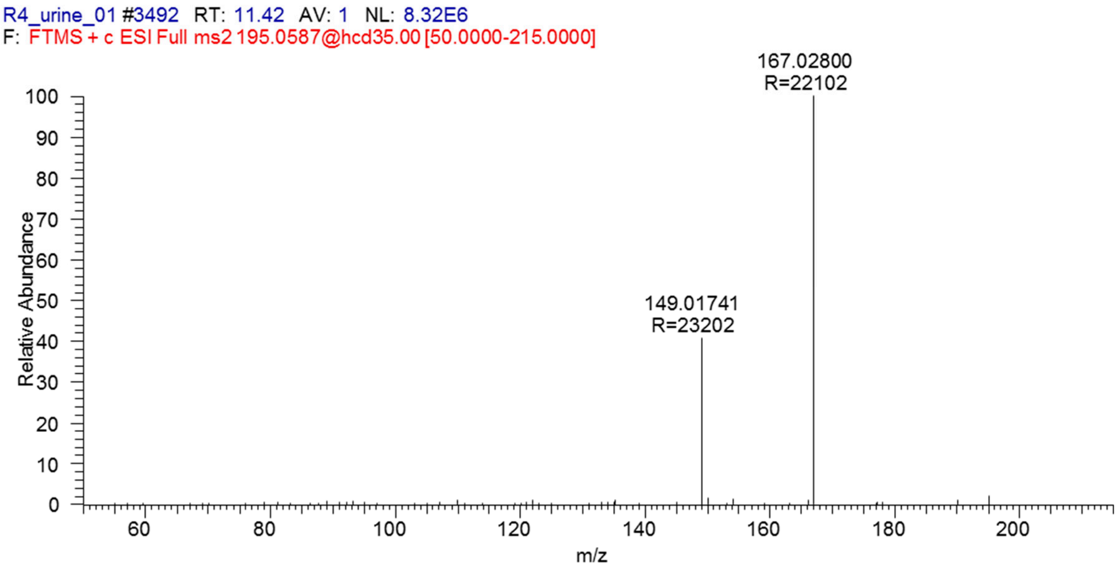

Figure S10. Mass-spectrum of M2b.

Table S5. Exact mass data for characteristic ions of M2b.

Compound	Parameter	Characteristic ions, *m/z*	
M2b	Theoretical *m/z*	167.0279	149.0173
	Experimental *m/z*	167.02800	149.01741
	Mass error, ppm	-0.6	-0.7

M3a,b

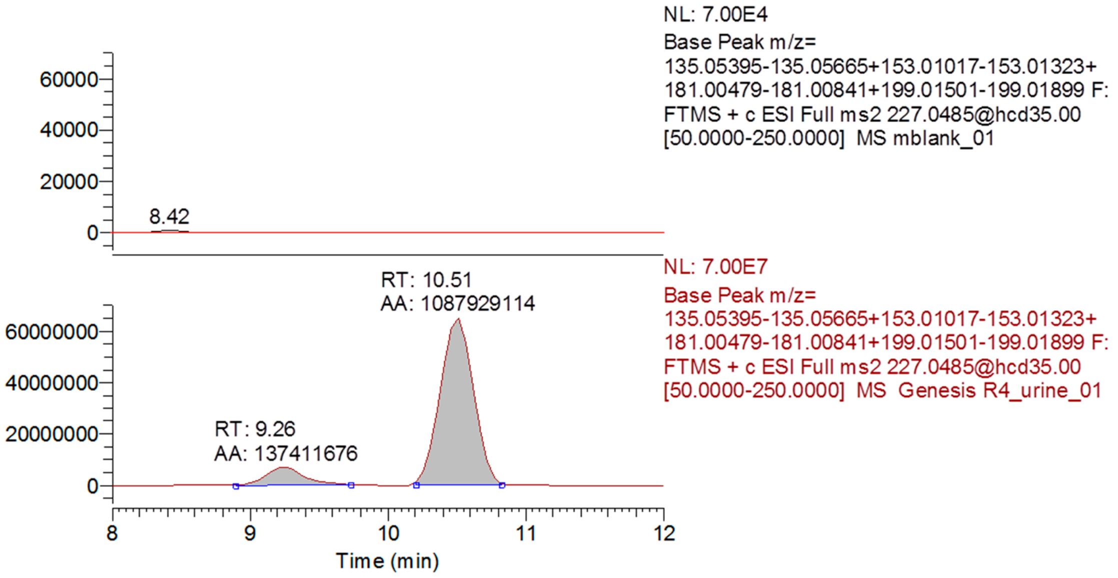

Figure S11. Reconstructed ion chromatogram of blank urine (top) and M3a and M3b (bottom) by product-ions (m/z) 199.0176, 181.0066, 153.0117, 135.0553.

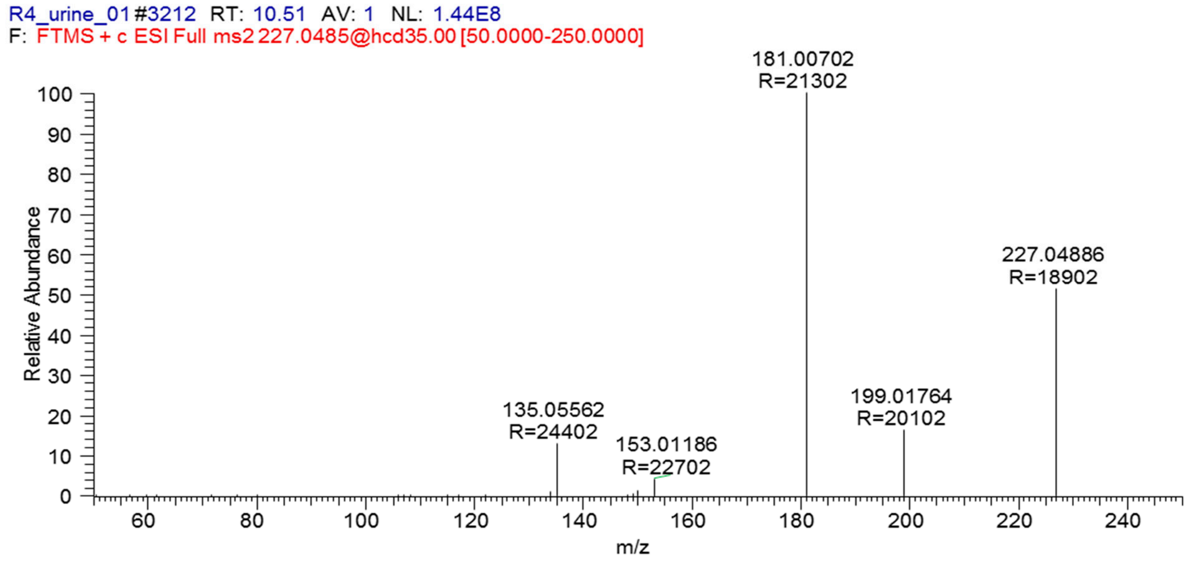

Figure S12. Mass-spectrum of M3(a,b). M3a and M3b has identical mass-spectrums.

Table S6. Exact mass data for characteristic ions of M3a,b.

Compound	Parameter	Characteristic ions, *m/z*				
M3(a,b)	Theoretical *m/z*	227.0486	199.0176	181.0066	153.0117	135.0553
	Experimental *m/z*	227.04886	199.01764	181.00702	153.01186	135.05562
	Mass error, ppm	-1.1	-0.2	-2.3	-1.0	-2.4

M4

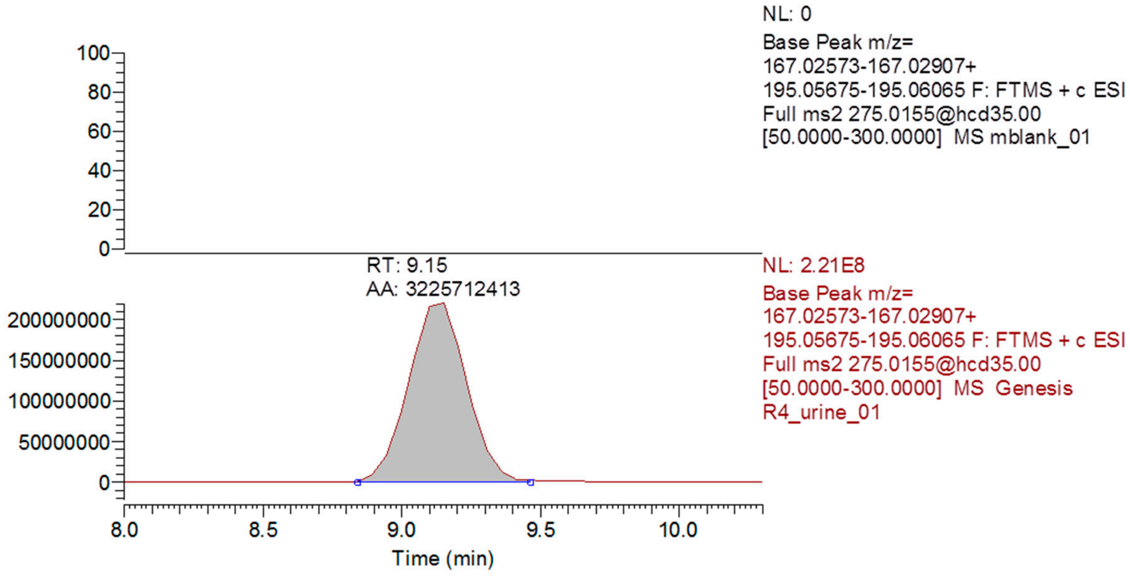

Figure S13. Reconstructed ion chromatogram of blank urine (top) and M4 (bottom) by product-ions (m/z) 195.0587, 167.0274.

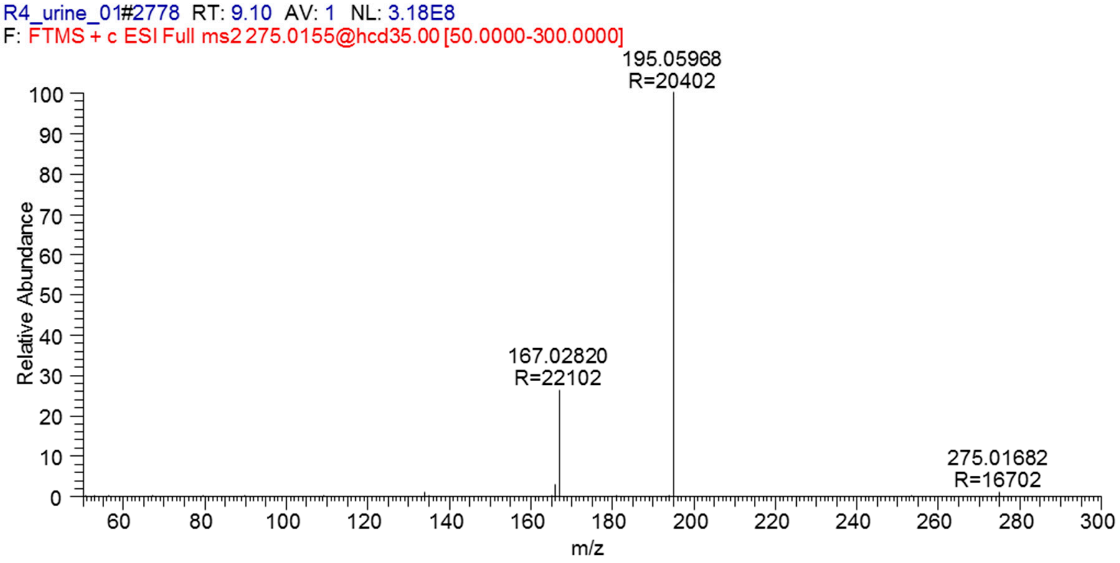

Figure S14. Mass-spectrum of M4.

Table S7. Exact mass data for characteristic ions of M4.

Compound	Parameter	Characteristic ions, *m/z*		
M4	Theoretical *m/z*	275.0155	195.0587	167.0274
	Experimental *m/z*	275.01682	195.05968	167.02820
	Mass error, ppm	-4.8	-5.0	-4.8

M5

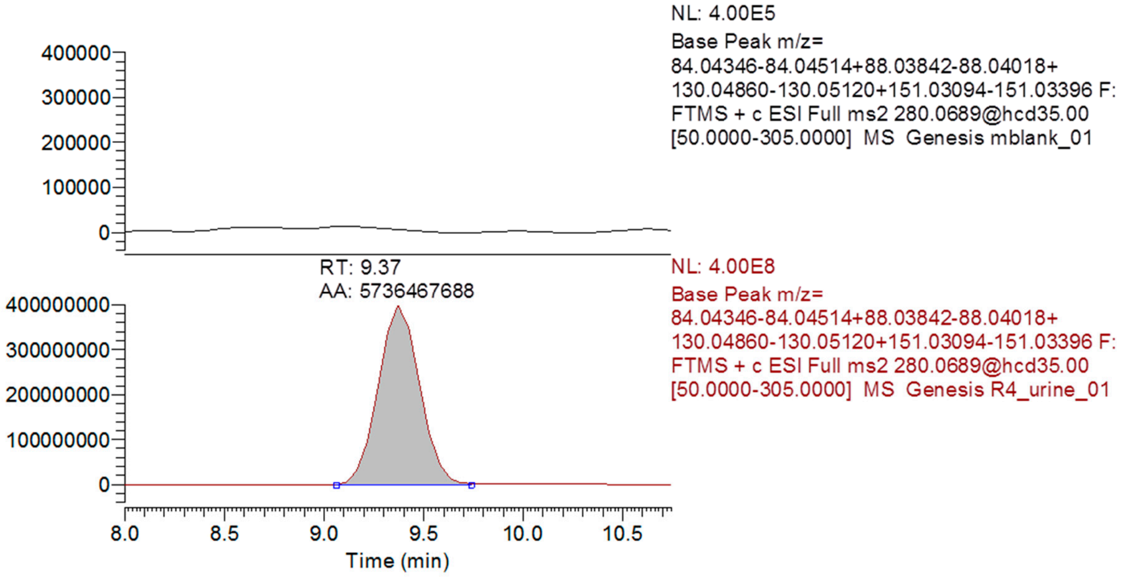

Figure S15. Reconstructed ion chromatogram of blank urine (top) and M5 (bottom) by product-ions (m/z) 151.0325, 130.0499, 88.0393, 84.0443.

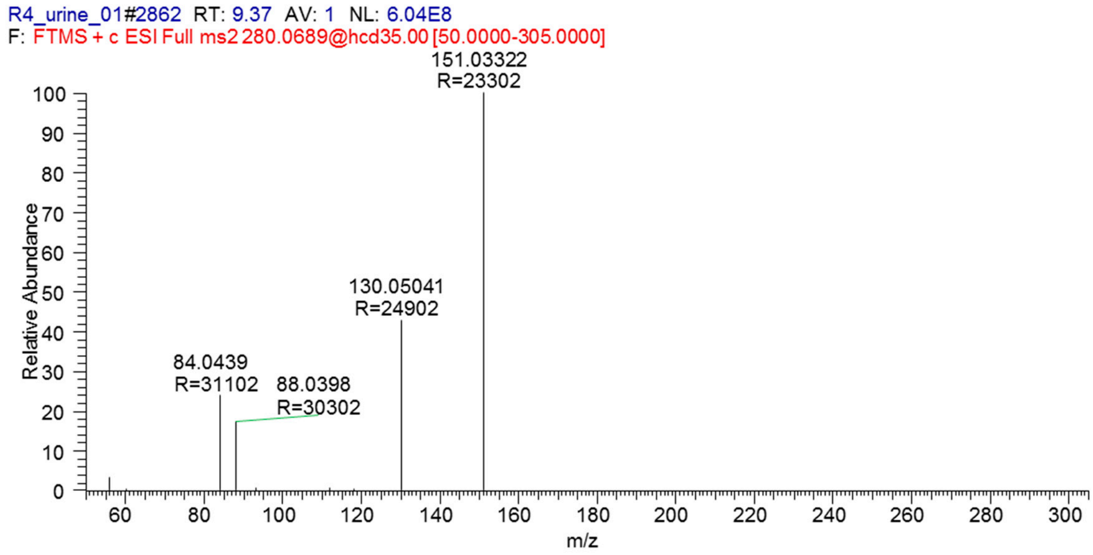

Figure S16. Mass-spectrum of M5.

Table S8. Exact mass data for characteristic ions of M5.

Compound	Parameter	Characteristic ions, *m/z*			
M5	Theoretical *m/z*	151.0325	130.0499	88.0393	84.0443
	Experimental *m/z*	151.03322	130.05041	88.0398	84.0439
	Mass error, ppm	-4.8	-3.9	-5.7	4.6

M6

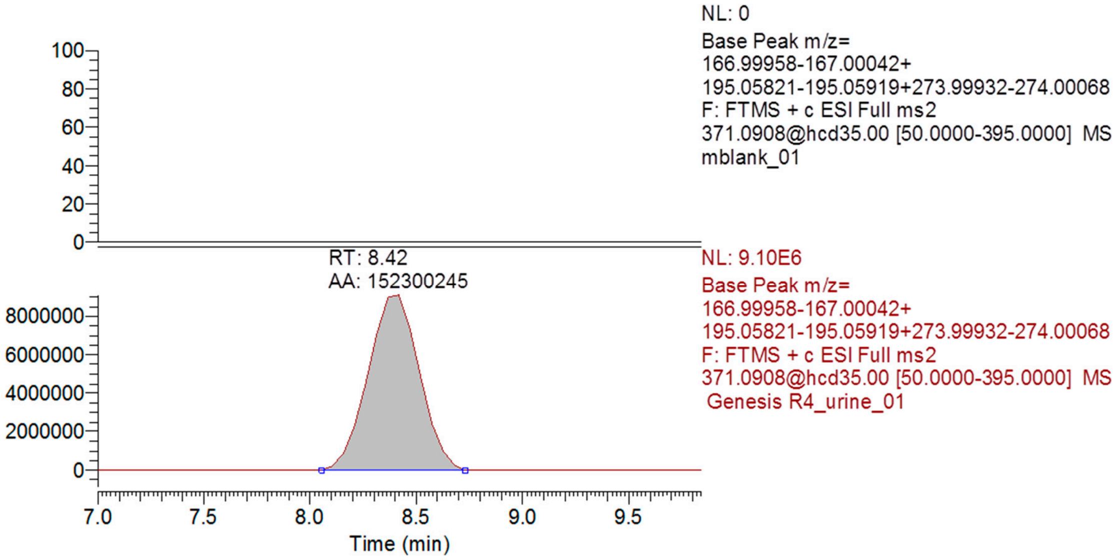

Figure S17. Reconstructed ion chromatogram of blank urine (top) and M6 (bottom) by product-ions (m/z) 195.0587, 167.0274.

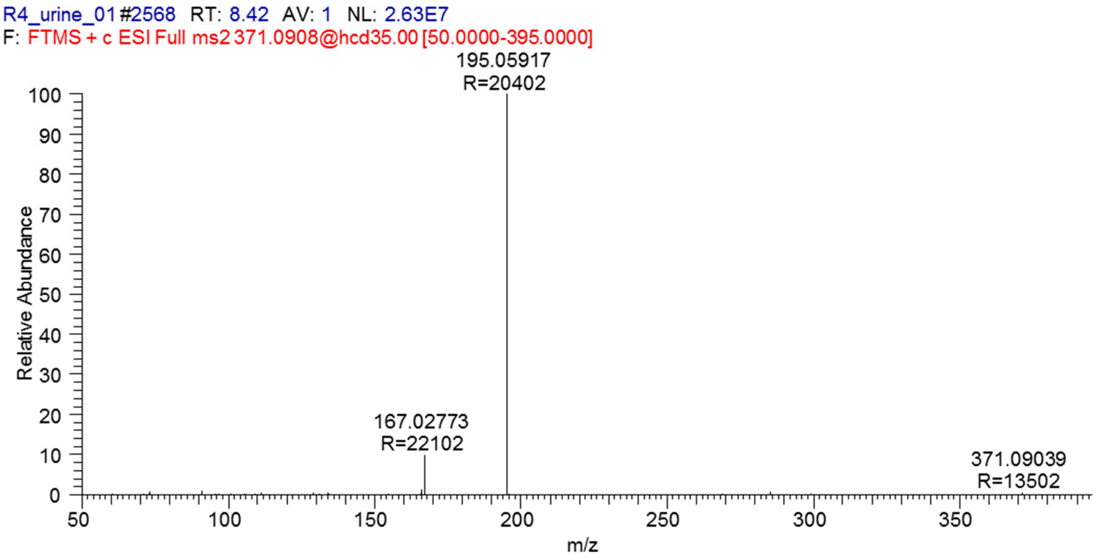

Figure S18. Mass-spectrum of M6.

Table S9. Exact mass data for characteristic ions of M6.

Compound	Parameter	Characteristic ions, *m/z*	
M6	Theoretical *m/z*	195.0587	167.0274
	Experimental *m/z*	195.05917	167.02773
	Mass error, ppm	-2.4	-2.0

In silico prediction of the Phase I metabolites of bemethyl using GLORY software tools

GLORY is the tool for predicting Phase 1metabolites in humans (de Bruyn Kops et al., 2019) based on the second generation Fast Metabolizer (FAME 2) algorithm (Šícho et al., 2019). FAME 2 is a machine learning model for the prediction of sites of metabolism (SoM) for drug-like and other xenobiotic compounds, which was developed using the extremely randomized trees algorithm and 2D circular descriptors of atoms and their environments (Šícho et al., 2017).

GLORY gives the score to the predicted metabolites indicating the probability of their formation: the higher score, the higher probability of the metabolite formation. GLORY score is calculated as the product of P and F, where *P* is the maximum SoM probability and F is the factor characterising whether the reaction type is common or not (reaction rules) (de Bruyn Kops et al., 2019).

The Figures S19-S23 and Tables S10-S14 represent the Phase I metabolites of bemethyl and its oxidation products according to GLORY software. MaxEfficiency Mode was used, which applies the reaction rules at all positions in the molecule regardless of the SoM probabilities.

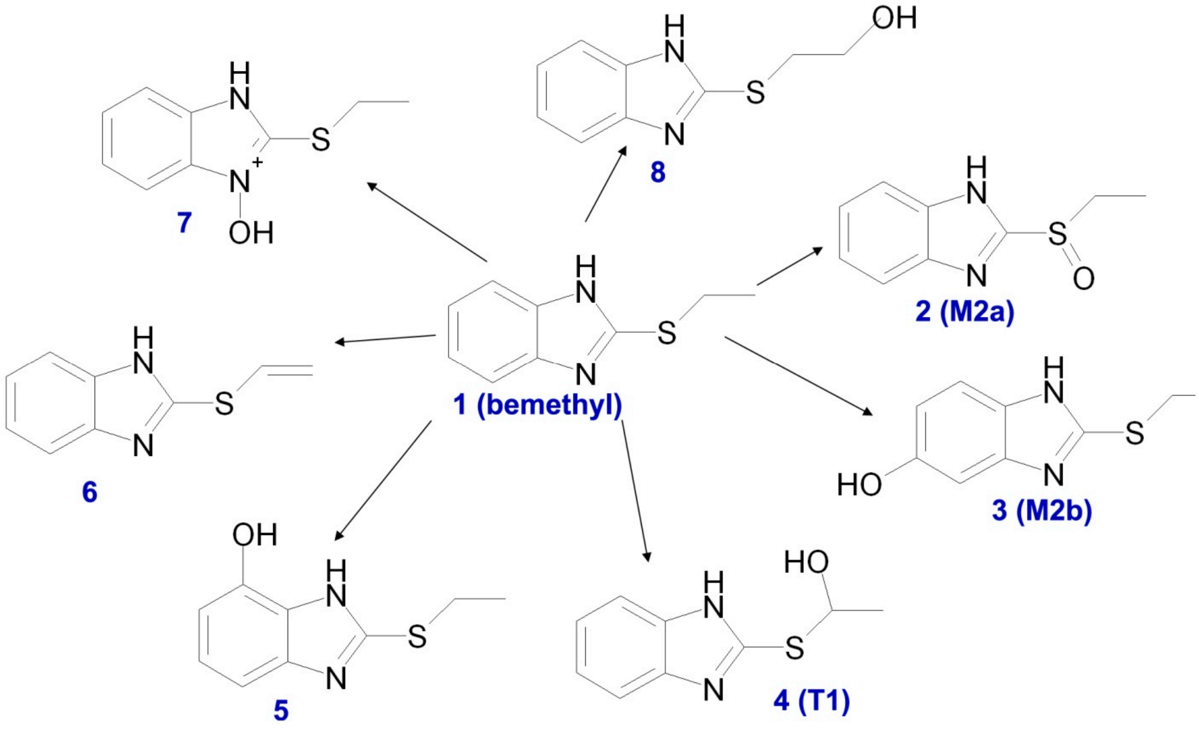

Figure S19. Bemethyl oxidation products according to GLORY software.

Table S10. Scores of GLORY software characterizing the probability of the formation of bemethyl (compound 1) oxidation products and Cytochrome P450 (CYP) isoforms participating in the oxidation reaction according to the BioTransformer.ca service

Input compound	Oxidation product (GLORY)	Score (GLORY)	CYP (BioTransformer.ca)
	**2 (M2a)**	4.20	CYP1A2, CYP2A6, CYP2B6, CYP2C9, CYP3A4
	**3 (M2b)**	2.49	CYP1A2, CYP2A6, CYP2B6, CYP2C9, CYP2C19, CYP3A4
	**4 (T1)**	2.11	ND
**1 (bemethyl)**	**5**	1.29	ND
	**6**	0.42	ND
	**7**	0.12	ND
	**8**	0.10	CYP1A2, CYP2A6, CYP2B6, CYP2C9, CYP2C19, CYP3A4

CYP, Cytochrome P450; ND, the metabolite has not been predicted by BioTransformer.ca

In the next step, we studied transformation of compounds 2 and 3, which are the most probable oxidations products of bemethyl (Figures S20, S21 and Tables S11, S12)

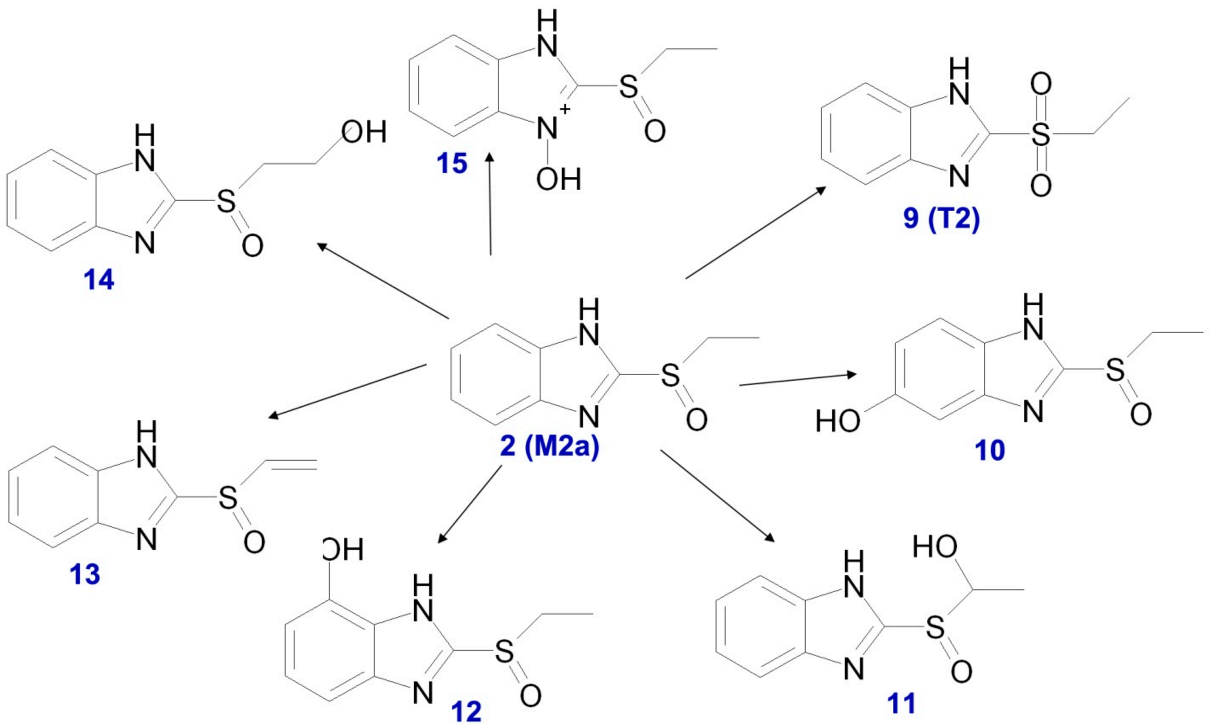

Figure S20. Oxidation products of bemethyl sulfoxide according to GLORY software

Table S11. Scores of GLORY software characterizing the probability of the formation of oxidation products of bemethyl sulfoxide (compound 2 and metabolite M2a) and Cytochrome P450 (CYP) isoforms participating in the oxidation reaction according to the BioTransformer.ca service

**Input compound**	**Oxidation product (GLORY)**	**Score (GLORY)**	**CYP (BioTransformer.ca)**
	**9 (T2)**	4.14	CYP2C9, CYP3A4
	**10**	2.54	CYP1A2, CYP2A6, CYP2C9, CYP2C19, CYP3A4
	**11**	1.78	ND
**2 (M2a)**	**12**	1.04	ND
	**13**	0.36	ND
	**14**	0.14	CYP1A2, CYP2A6, CYP2C9, CYP2C19, CYP3A4
	**15**	0.14	ND

CYP, Cytochrome P450; ND, the metabolite has not been predicted by BioTransformer.ca

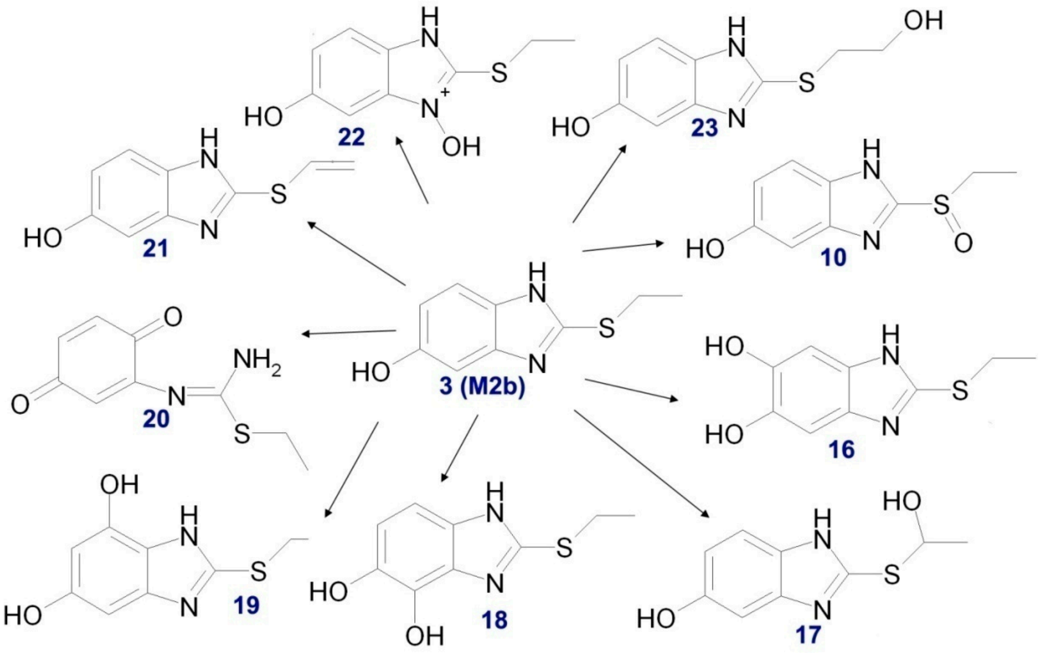

Figure S21. Oxidation products of hydroxyl bemethyl according to GLORY software

Table S12. Scores of GLORY software characterizing the probability of the formation of oxidation products of hydroxyl bemethyl (compound **3** and metabolite M2b) and Cytochrome P450 (CYP) isoforms participating in the oxidation reaction according to the BioTransformer.ca service

Input compound	Oxidation product (GLORY)	Score (GLORY)	CYP (BioTransformer.ca)
	**10**	3.87	CYP1A2, CYP2C9, CYP2D6, CYP3A4
	**16**	2.15	CYP1A2, CYP2C9, CYP2C19, CYP2D6, CYP3A4
	**17**	1.99	ND
	**18**	1.86	ND
**3 (M2b)**	**19**	0.58	ND
	**20**	0.43	ND
	**21**	0.40	ND
	**22**	0.14	ND
	**23**	0.06	CYP1A2, CYP2C9, CYP2C19, CYP2D6, CYP3A4

CYP, Cytochrome P450; ND, the metabolite has not been predicted by BioTransformer.ca

Compounds 9, 10 and 16 are the most probable oxidation products of compounds 2 and 3. In the final step, we studied their transformation (Figures S22, S23 and Tables S13, S14).

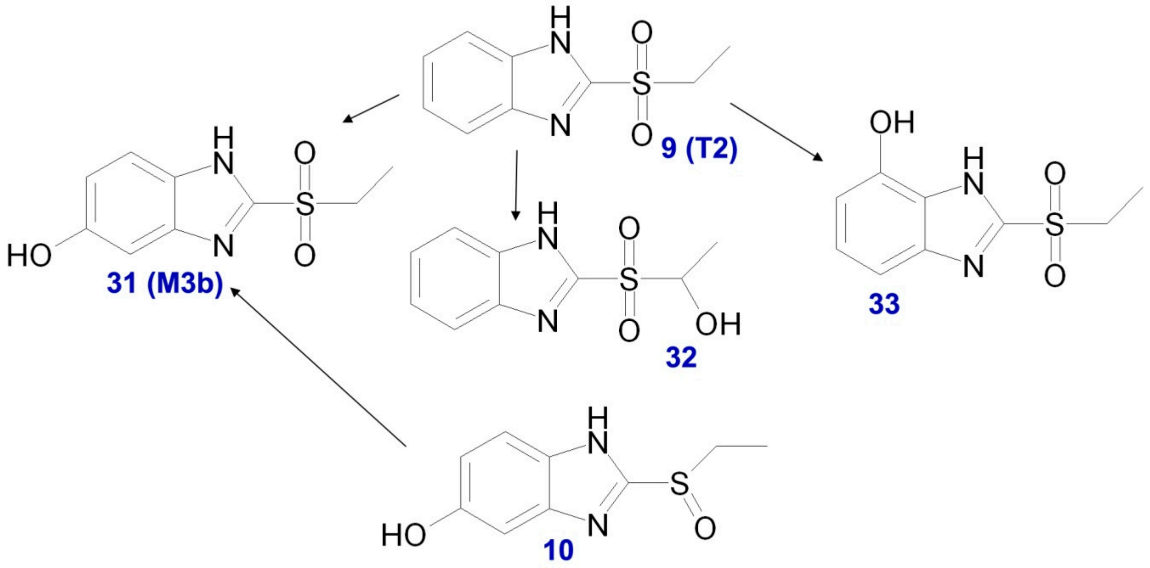

Figure S22. Oxidation products of bemethyl sulfone and hydroxyl bemethyl sulfoxide according to GLORY software (only the most probable products are shown)

Table S13. Scores of GLORY software characterizing the probability of the formation of oxidation products of bemethyl sulfone (compound 9 and transitional metabolite T2) and hydroxyl bemethyl sulfoxide (compound 10) and Cytochrome P450 (CYP) isoforms participating in the oxidation reaction according to the BioTransformer.ca service.

Input compound	Oxidation product (GLORY)	Score (GLORY)	CYP (BioTransformer.ca)
**9 (T2)**	**24 (M3b)**	2.52	CYP1A2, CYP2C9, CYP2C19, CYP3A4
	**25**	1.53	ND
	**26**	1.01	ND
**10**	**24 (M3b)**	3.87	CYP2C9, CYP3A4

CYP, Cytochrome P450; ND, the metabolite has not been predicted by BioTransformer.ca

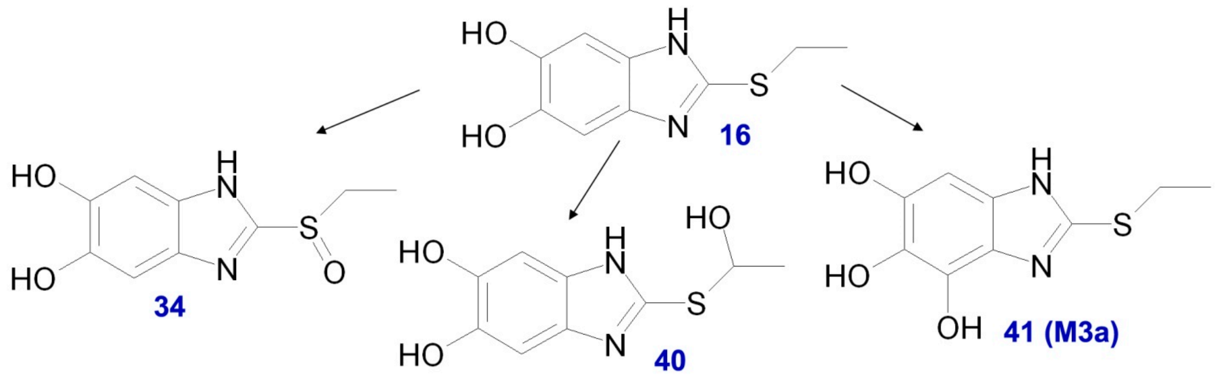

Figure S23. Oxidation products of dihydroxybemethyl according to GLORY software (only the most probable products are shown).

Table S14. Scores of GLORY software characterizing the probability of the formation of oxidation products of dihydroxybemethyl (compound 16) and Cytochrome P450 (CYP) isoforms participating in the oxidation reaction according to the BioTransformer.ca service

Input compound	Oxidation product (GLORY)	Score (GLORY)	CYP (BioTransformer.ca)
	**27**	3.88	CYP1A2, CYP2C9, CYP2D6, CYP3A4
**16**	**28**	1.98	ND
	**29 (M3a)**	1.81	ND

References for Supplementary

de Bruyn Kops, C.; Stork, C.; Šícho, M.; Kochev, N.; Svozil, D.; Jeliazkova, N.; Kirchmair, J. GLORY: Generator of the Structures of Likely Cytochrome P450 Metabolites Based on Predicted Sites of Metabolism. *Front. Chem.* **2019**, *7*, 402. doi: 10.3389/fchem.2019.00402.

Šícho, M.; Stork, C.; Mazzolari, A.; de Bruyn Kops, C.; Pedretti, A.; Testa, B.; Vistoli, G.; Svozil, D.; Kirchmair, J. FAME 3: Predicting the Sites of Metabolism in Synthetic Compounds and Natural Products for Phase 1 and Phase 2 Metabolic Enzymes. *J. Chem. Inf. Model.* **2019**, *59(8)*, 3400–3412. doi: 10.1021/acs.jcim.9b00376.

Šícho, M.; de Bruyn Kops, C.; Stork, C.; Svozil, D.; Kirchmair, J. FAME 2: Simple and Effective Machine Learning Model of Cytochrome P450 Regioselectivity. *J. Chem. Inf. Model* **2017**, *57(8)*, 1832–1846. doi: 10.1021/acs.jcim.7b00250.
